# Relationship between muscle metabolic rate and muscle torque complexity during fatiguing intermittent isometric contractions in humans

**DOI:** 10.14814/phy2.14240

**Published:** 2019-09-25

**Authors:** Jamie Pethick, Samantha L. Winter, Mark Burnley

**Affiliations:** ^1^ Endurance Research Group School of Sport and Exercise Sciences University of Kent Canterbury United Kingdom

**Keywords:** Fatigue, muscle, metabolic rate, nonlinear dynamics

## Abstract

To test the hypothesis that a system’s metabolic rate and the complexity of fluctuations in the output of that system are related, thirteen healthy participants performed intermittent isometric knee extensor contractions at intensities where a rise in metabolic rate would (40% maximal voluntary contraction, MVC) and would not (20% MVC) be expected. The contractions had a 60% duty factor (6 sec contraction, 4 sec rest) and were performed until task failure or for 30 min, whichever occurred sooner. Torque and surface EMG signals were sampled continuously. Complexity and fractal scaling of torque were quantified using approximate entropy (ApEn) and the detrended fluctuation analysis (DFA) α scaling exponent. Muscle metabolic rate was determined using near‐infrared spectroscopy. At 40% MVC, task failure occurred after (mean ± SD) 11.5 ± 5.2 min, whereas all participants completed 30 min of contractions at 20% MVC. Muscle metabolic rate increased significantly after 2 min at 40% MVC (2.70 ± 1.48 to 4.04 ± 1.23 %·s^‐1^, *P* < 0.001), but not at 20% MVC. Similarly, complexity decreased significantly at 40% MVC (ApEn, 0.53 ± 0.19 to 0.15 ± 0.09; DFA α, 1.37 ± 0.08 to 1.60 ± 0.09; both *P* < 0.001), but not at 20% MVC. The rates of change of torque complexity and muscle metabolic rate at 40% MVC were significantly correlated (ApEn, *ρ* = −0.63, *P* = 0.022; DFA, *ρ* = 0.58, *P* = 0.037). This study demonstrated that an inverse relationship exists between muscle torque complexity and metabolic rate during high‐intensity contractions.

## Introduction

Life, according to Macklem (2009), can only exist in a phase transition between order and chaos, wherein a dynamic balance between stability and adaptability exists. This balance results in constant fluctuations in physiological time‐series (such as heart rate, gait and muscle torque; Lipsitz and Goldberger, 1992; Goldberger et al., 2002a), which do not simply represent an error signal in homeostatic control. Recent evidence has suggested that the structure or “complexity” of these fluctuations directly reflects the adaptability the system of origin possesses (Seely and Macklem, 2012). It is important to distinguish between the complexity of these fluctuations and the more traditionally assessed variability. The latter represents the amplitude of fluctuations (often reported as the standard deviation or coefficient of variation), whilst the former identifies the temporal structures within a time‐series (Goldberger et al., 2002a). The complex outputs exhibited by physiological systems are characterised by a number of properties that traditional variability statistics cannot quantify; namely, temporal irregularity, time irreversibility and long‐range (fractal) correlations (Pincus, 1991; Lipsitz and Goldberger, 1992; Goldberger et al., 2002a). Thus, the temporal structures within physiological time‐series contain information additional to, and distinct from, amplitude‐based measures of variability (Lipsitz and Goldberger, 1992). Complexity is quantified using algorithms drawn from information theory, such as approximate entropy (ApEn; Pincus, 1991), which quantifies the irregularity of a system’s output, and from fractal geometry, such as detrended fluctuation analysis (DFA; Peng et al., 1994), which quantifies the long‐range correlations present in an output and differentiates outputs that are random (white noise), statistically self‐similar (pink or 1/f noise) or Brownian in nature. It is important to note that reliance on single metrics for complexity analysis can be misleading, as white noise is irregular (producing high values for ApEn) without being physiologically complex (Goldberger et al., 2002b). For this reason, we quantify complexity using a regularity statistic (ApEn) and a statistic quantifying the signal’s fractal scaling properties (DFA).

Complex outputs arise from the interaction of multiple structural units and feedback loops that operate over a range of temporal and spatial scales (Lipsitz and Goldberger, 1992; Goldberger et al., 2002a). In the context of the neuromuscular system, ensembles of interconnected components, such as motor cortical neurones, spinal motoneurons, muscles fibers and muscle afferents, interact to produce complex patterns of force/torque output (Vaillancourt and Newell, 2003; Forrest et al., 2014). Reduced muscle torque complexity has been frequently observed with aging (Vaillancourt and Newell, 2003) and disease states (Vaillancourt et al., 2001), with each case representing a loss of neuromuscular system adaptability. We have recently extended this loss of muscle torque complexity from aging and disease to acute neuromuscular fatigue (Pethick et al., 2015,2016,2018a,2018b). Specifically, both maximal and submaximal intermittent isometric contractions of the knee extensors have been demonstrated to result in a progressive loss of muscle torque complexity, as measured by a decrease in ApEn (indicating increased regularity) and an increase in the DFA α scaling exponent (indicating a move toward more Brownian noise).

Because life is an open thermodynamic system, whose function depends fundamentally on energy transfer processes, Macklem (2009) and Seely and Macklem (2012) have hypothesized that the complexity of any physiological output depends on the metabolic rate of the system producing it. In other words, a system is at its most adaptable, and thus most complex, when operating at a low fraction of its maximum metabolic rate (Seely and Macklem, 2012). Increasing the relative demand on a system (either by increasing its operating metabolic rate, or by reducing the maximal attainable metabolic rate), should predictably decrease system complexity. However, this hypothesis has yet to be directly tested in any physiological system. Testing it requires a system whose output fluctuates in a complex fashion and in which metabolic rate can vary widely, be manipulated experimentally, and measured accurately *in vivo*. The neuromuscular system meets each of these requirements: isometric muscle torque output possesses complex fluctuations (Vaillancourt and Newell, 2003; Forrest et al., 2014), with this complexity reduced by high‐intensity fatiguing exercise (Pethick et al., 2015, 2016,2018a,2018b); muscle metabolic rate can increase more than 20‐fold from rest to high‐intensity exercise; and muscle metabolic rate can be measured noninvasively using near‐infrared spectroscopy (NIRS; Van Beekvelt et al., 2001; Russell et al., 2002). By periodically cuffing the thigh, thus occluding arterial inflow, NIRS can be used to quantify the rate of rise in deoxygenated hemoglobin/myoglobin (HHb), which is directly proportional to the muscle oxygen consumption ($m\dot{V}O_{2}$; Van Beekvelt et al., 2001; De Ruiter et al., 2005).

Muscle metabolic rate can be increased by increasing the torque or power output requirements, or by performing constant‐load high‐intensity exercise, in which the torque or power output demands remain constant, but metabolic rate progressively increases until task failure, consequent to the slow component of oxygen uptake kinetics (Vøllestad et al., 1990; see Whipp 1994 for review). In the present experiments, participants performed low‐intensity isometric contractions (at 20% of the maximal voluntary contraction [MVC] torque) intended to result in a relatively low and steady‐state $m\dot{V}O_{2}$, and high‐intensity contractions (at 40% MVC) intended to result in a high and nonsteady state $m\dot{V}O_{2}$. If $m\dot{V}O_{2}$ and system complexity are related, as hypothesized, then at 20% MVC neither $m\dot{V}O_{2}$ nor complexity should change as a function of time, whereas both should change systematically at 40% MVC, with the increase in muscle metabolic rate being accompanied by a proportional decrease in muscle torque complexity. The above relationships are thought to relate to the relative metabolic rate of the tissue or organ of interest (i.e., the fraction of maximal O_2_ uptake, $\dot{V}O_{\text{2max}}$), not the absolute metabolic rate. For this reason, we tested these relationships using $m\dot{V}O_{2}$ calculated from the percentage of the maximal HHb signal in a repeated measures design (see Materials and Methods); these relationship should not be expected to hold between participants (e.g., sedentary controls vs. athletes), or between different tissues or organ systems, each of which have their own respective maximal metabolic rates.

The purpose of this study was, therefore, to investigate the hypothesized inverse relationship between complexity and metabolic rate, by using two contraction intensities in which a rise in $m\dot{V}O_{2}$ beyond the initial transient would (40% MVC) and would not be expected (20% MVC). The specific experimental hypotheses tested were that high‐intensity contractions (at 40% MVC) would be accompanied by a progressive rise in $m\dot{V}O_{2}$ and a progressive loss of muscle torque complexity, quantified by a decrease in ApEn and an increase in the DFA α scaling exponent; while low‐intensity contractions (at 20% MVC) would result in the attainment of steady states in both $m\dot{V}O_{2}$ and muscle torque complexity.

## Materials and Methods

### Participants

Thirteen healthy participants (10 male, 3 female; mean ± SD: age 27.6 ± 6.4 years; height 1.75 ± 0.08 m; body mass 71.4 ± 9.0 kg) provided written informed consent to participate in the study, which was approved by the ethics committee of the University of Kent, and which adhered to the Declaration of Helsinki. The participants represented a convenience sample, the size of which was based on that of our previous experiments investigating torque complexity (Pethick et al., 2015, 2016). Participants were instructed to arrive at the laboratory in a rested state (having performed no strenuous exercise in the preceding 24 h) and to have consumed neither any food nor caffeinated beverages in the 3 h before arrival. Participants attended the laboratory at the same time of day (±2 h) during each visit.

### Experimental design

Participants were required to visit the laboratory on three occasions, with a minimum of 48 h between each visit. During their first visit, participants were familiarized with all testing equipment and procedures, and the settings for the dynamometer and stimulator were recorded. During the next two visits, participants performed, in a randomized order, high‐intensity (40% MVC) and low‐intensity (20% MVC) intermittent isometric knee extension contractions to task failure or for 30 min, whichever occurred sooner. In each trial, torque output was sampled continuously to allow the quantification of complexity, muscle activity was measured using the vastus lateralis electromyogram (EMG), muscle oxygen consumption was measured using NIRS, and MVCs with supramaximal femoral nerve stimulation were used to quantify global, central and peripheral fatigue.

### Dynamometry

During all visits, participants were seated in the chair of a Cybex isokinetic dynamometer (HUMAC Norm; CSMi, Massachusetts, USA), initialized and calibrated according to the manufacturer’s instructions. Their right leg was attached to the lever arm of the dynamometer, with the seating position adjusted to ensure that the lateral epicondyle of the femur was in line with the axis of rotation of the lever arm. Participants sat with relative hip and knee angles of 85° and 90°, respectively, with full extension being 0°. The lower leg was securely attached to the lever arm above the malleoli with a padded Velcro strap, while straps secured firmly across both shoulders and the waist prevented any extraneous movement and the use of the hip extensors during the isometric contractions. The seating position was recorded during the first visit and replicated during each subsequent visit.

### Femoral nerve stimulation

Electrical stimulation of the femoral nerve was used to assess neuromuscular fatigue processes, in the same way as described by Pethick et al. (2015). The anode, a carbon rubber electrode with adhesive gel (100 x 50 mm; Phoenix Healthcare Products Ltd., Nottingham, UK), was placed lateral to the ischial tuberosity, on the posterior aspect of the leg. The position of the cathode was established using a motor point pen (Compex; DJO Global, Guildford, UK), and determined based on the location in the femoral triangle giving the largest twitch and greatest peak‐to‐peak amplitude of the compound muscle action potential (M‐wave) following single stimulation at 100 mA, using a constant‐current variable voltage stimulator (Digitimer DS7AH, Welwyn Garden City, UK). Following establishment of the precise cathode location, an Ag/AgCl electrode (32 x 32 mm; Nessler Medizintechnik, Innsbruck, Austria) coated in conductive gel was placed over the femoral nerve.

The appropriate stimulator current was then established by incrementally increasing the current (in steps of 20 mA) until knee extensor torque and the M‐wave response to single twitches had plateaued. This was verified with stimulation delivered during a contraction at 50% MVC to ensure a maximal M‐wave during an isometric contraction was also evident. Once this was obtained, the stimulator current was then increased to 130% of the current producing a maximal M‐wave. In all subsequent trials, doublet stimulation (two 200 µsec pulses with 10 msec interpulse interval) was used.

### Surface EMG

The EMG of the vastus lateralis was sampled using Ag/AgCl electrodes (32 x 32 mm; Nessler Medizintechnik, Innsbruck, Austria). Prior to attachment of the electrodes, the skin of the participants was shaved, abraded and then cleaned with an alcohol swab over the belly of the muscle, in order to reduce impedance. The electrodes were placed on the prepared skin over the belly of the muscle in a direction parallel to the alignment of the muscle fibers. A reference electrode was placed on prepared skin medial to the tibial tuberosity. Care was taken to ensure that the electrode locations were identical between sessions. The raw EMG signals were sampled at 1 kHz, amplified (gain 1000; Biopac MP150; Biopac Systems Inc., California, USA) and band‐pass filtered (10–500 Hz; Biopac MP 150; Biopac Systems Inc., California, USA).

### Muscle oxygen consumption

$m\dot{V}O_{2}$ from the vastus lateralis was obtained using a continuous‐wave NIRS device (Oxymon Mk III, Artinis Medical Systems, Netherlands), calibrated according to the manufacturer’s instructions before each trial. The NIRS device generated light at three wavelengths (905, 850 and 770 nm) corresponding to the absorption wavelengths of oxyhemoglobin (O_2_Hb) and deoxyhemoglobin (HHb). An area at the level of the largest circumference of the vastus lateralis was shaved, abraded and cleaned with an alcohol swab. The NIRS optode was then placed at this location and secured with biadhesive tape, such that the optode did not move during contraction. A blood pressure cuff (Hokanson E20 cuff inflator; D.E. Hokanson Inc., Bellevue, USA) was placed proximal to the NIRS optode, and was used to maintain blood volume under the optode during measurement. NIRS data were collected at 10 Hz. Adipose tissue thickness at the site of measurement was assessed, as per the recommendations of Ferrari et al. (2011), using skinfold callipers. However, because the HHb and O_2_Hb signals were normalized using an ischemic calibration rather than calculating absolute concentration changes, adipose tissue thickness did not influence $m\dot{V}O_{2}$ (see below, Ryan et al., 2012).

### Protocol

All visits followed a pattern of data acquisition similar to that which we have previously reported (Pethick et al., 2015, 2016), with the addition of $m\dot{V}O_{2}$ measurements. The pattern of data acquisition was identical for the 20% MVC and 40% MVC trials. Briefly, each trial consisted of instrumentation and femoral nerve stimulation set up, followed by the measurement of MVCs and the resting $m\dot{V}O_{2}$. After this, the exercise trial itself was performed for 30 min or to task failure. The $m\dot{V}O_{2}$ during the exercise trial was established at the end of each minute by brief occlusions. Task end/failure was followed immediately by an MVC. Lastly, a resting 3–5 min occlusion was performed to normalize the NIRS data. The detail and timings of each of these measurements is given below.

Each visit began with the instrumentation of the participants and the establishment of the correct dynamometer seating position and supramaximal stimulation response. Participants then performed a series of brief (3 sec) MVCs to establish their maximum torque. These contractions were separated by a minimum of 60 sec rest, and continued until the peak torques in three consecutive contractions were within 5% of each other. Participants were given a countdown, followed by very strong verbal encouragement to maximize torque. The first MVC was used to establish the fresh maximal EMG signal, against which the subsequent EMG signals were normalized (*Data analysis*; see below). The second and third MVCs were performed with femoral nerve stimulation delivered during the contraction and at rest after the contraction. The stimulation during the contraction was delivered during a plateau in maximal torque, in order to test the maximality of the contraction and provide the resting voluntary activation; while the stimulation at rest was delivered 2 sec after the contraction, in order to establish the fresh potentiated doublet torque. All subsequent contractions with femoral nerve stimulation were conducted in this manner.

After the establishment of maximum torque, the resting $m\dot{V}O_{2}$ of the vastus lateralis was assessed based on the decrease in muscle oxygenation which accompanies an arterial occlusion (Ryan et al., 2012, 2013). For this, a blood pressure cuff was rapidly inflated to 300 mmHg using a Hokanson AG101 (D.E. Hokanson Inc., Bellevue, USA). Four resting measurements were made using 10 sec of arterial occlusion, each separated by 60 sec. The resting $m\dot{V}O_{2}$ was calculated using linear regression with the first 8 sec of each occlusion (*Data analysis*; see below). Participants then rested for 10 min before performing the experimental trial.

### Experimental trials

Participants performed intermittent isometric knee extension contractions at either 20% or 40% MVC, in a randomized order. The target torques were based on the highest instantaneous torque recorded during the MVCs in the first experimental visit. The duty cycle for the contractions was 60%; with contractions held for 6 sec and being followed by 4 sec rest. Participants were instructed to match their instantaneous torque with a target bar superimposed on the display in front of them and were required to continue matching this torque for as much of the 6 sec contraction as possible. The contractions continued for 30 min or until task failure, whichever occurred sooner. Task failure was defined as the point at which the participants failed to reach the target torque on three consecutive contractions, despite strong verbal encouragement. Participants were not informed of the elapsed time during the trials, but were informed of each “missed” contraction. At task end or after the third missed contraction, participants were instructed to immediately produce an MVC, which was accompanied by femoral nerve stimulation.

At the end of each minute (i.e., after every fifth contraction), $m\dot{V}O_{2}$ was assessed. The blood pressure cuff was rapidly inflated to 300 mmHg for 5 sec, with $m\dot{V}O_{2}$ calculated using linear regression over the course of this occlusion. This measure of $m\dot{V}O_{2}$ was performed instead of a targeted contraction. Here, $m\dot{V}O_{2}$ was also assessed immediately prior to the MVC performed at task end/failure. Five minutes after task end/failure, an ischemia/hyperemia calibration was performed to normalize the NIRS signals. The blood pressure cuff was inflated to 300 mmHg for 3–5 min (or until the NIRS signals plateaued). This deoxygenated the tissue under the optode (i.e., 0% oxygenation), while the peak hyperemic response upon release of the cuff represented 100% oxygenation.

### Data acquisition and participant interface

Data acquisition was performed in a similar manner as described in Pethick et al. (2015). Briefly, the isokinetic dynamometer, stimulator and EMG were connected via BNC cables to a Biopac MP150 (Biopac Systems Inc., California, USA) and a CED Micro 1401‐3 (Cambridge Electronic Design, Cambridge, UK) interfaced with a personal computer. These data were sampled at 1 kHz and collected in Spike2 (Version 7; Cambridge Electronic Design, Cambridge, UK). The NIRS data were sampled at 10 Hz and collected in OxySoft (Artinis Medical Systems, Netherlands).

A chart containing the instantaneous torque was projected onto a screen placed ~ 1 m in front of the participant. A scale consisting of a thin line (1 mm thick) was superimposed on the torque chart and acted as a target, so that participants were able to match their instantaneous torque output to the target torque during each visit.

### Data analysis

All data were analyzed using code written in MATLAB R2013a (The MathWorks, Massachusetts, USA). The data analysis focused on four specific areas: (1) basic measures of torque and EMG; (2) measures of central and peripheral fatigue; (3) the variability and complexity of torque output; and (4) measures of muscle oxygen consumption ($m\dot{V}O_{2}$).

#### Torque and EMG

The mean and peak torque for each contraction in both trials were determined. The mean torque was calculated based on the steadiest 5 sec of each contraction, with MATLAB code identifying the 5 sec of each contraction with the lowest standard deviation. The point of task failure was determined as in Pethick et al. (2015). The mean torque produced during the first five contractions was calculated, with task failure deemed to occur when the mean torque recorded during three consecutive contractions was more than 5 N·m below the mean torque of the first five contractions, with the first of these contractions being considered the point of task failure.

The EMG output from the vastus lateralis for each contraction was full‐wave rectified during each 5 sec window. The average rectified EMG (arEMG) was then calculated and normalized by expressing the arEMG as a fraction of the mean arEMG obtained during a 3 sec MVC from the fresh muscle performed at the beginning of the trial.

#### Central and peripheral fatigue

Measures of central and peripheral fatigue were calculated based on the stimuli delivered during and after the MVCs performed pre‐test and at task end/failure. Peripheral fatigue was evidenced by a fall in the potentiated doublet torque. Central fatigue was evidenced by a decline in voluntary activation, quantified using the twitch interpolation technique (Belanger and McComas, 1981; Behm et al., 1996):

(1) Voluntary activation(%)=1-(superimposed doublet/resting doublet)×100

where the superimposed doublet was that measured during the contraction of interest and the potentiated doublet was measured at rest 2 sec after that contraction.

#### Variability and complexity

All measures of variability and complexity were calculated using the steadiest 5 sec of each contraction, identified by MATLAB as the 5 sec containing the lowest standard deviation (SD). The amount of variability in the torque output of each contraction was measured using the SD and coefficient of variation (CV). The SD provides a measure of the absolute amount of variability in a time series, while the CV provides a measure of the amount of variability in a time‐series normalized to the mean of the time series.

The temporal structure, or complexity, of torque output was examined using multiple time domain analyses, as recommended by Goldberger et al. (2002a,2002b). The regularity of torque output was determined using approximate entropy (ApEn; Pincus, 1991) and the temporal fractal scaling of torque was estimated using the detrended fluctuation analysis (DFA; Peng et al., 1994) α scaling exponent. The calculations of ApEn and DFA are detailed in Pethick et al. (2015). In brief, ApEn was calculated with the template length, *m*, set at 2 and the tolerance, *r*, set at 10% of the SD of torque output, and DFA was calculated across time scales (57 boxes ranging from 1250 to 4 data points). In two participants in the 40% MVC condition, a degree of crossover Hu et al. (2001) was identified in the log–log plot of fluctuation size versus box size (as shown by an *r* < 0.95). To account for this effect, an iterative piecewise least squares linear regression was used to fit two lines to the plot for these two trials, and two α exponents were quantified. The second of these (α_2_, representing longer, physiologic timescales) was used in the DFA α exponent analysis.

#### Muscle oxygen consumption

**Figure 1:**
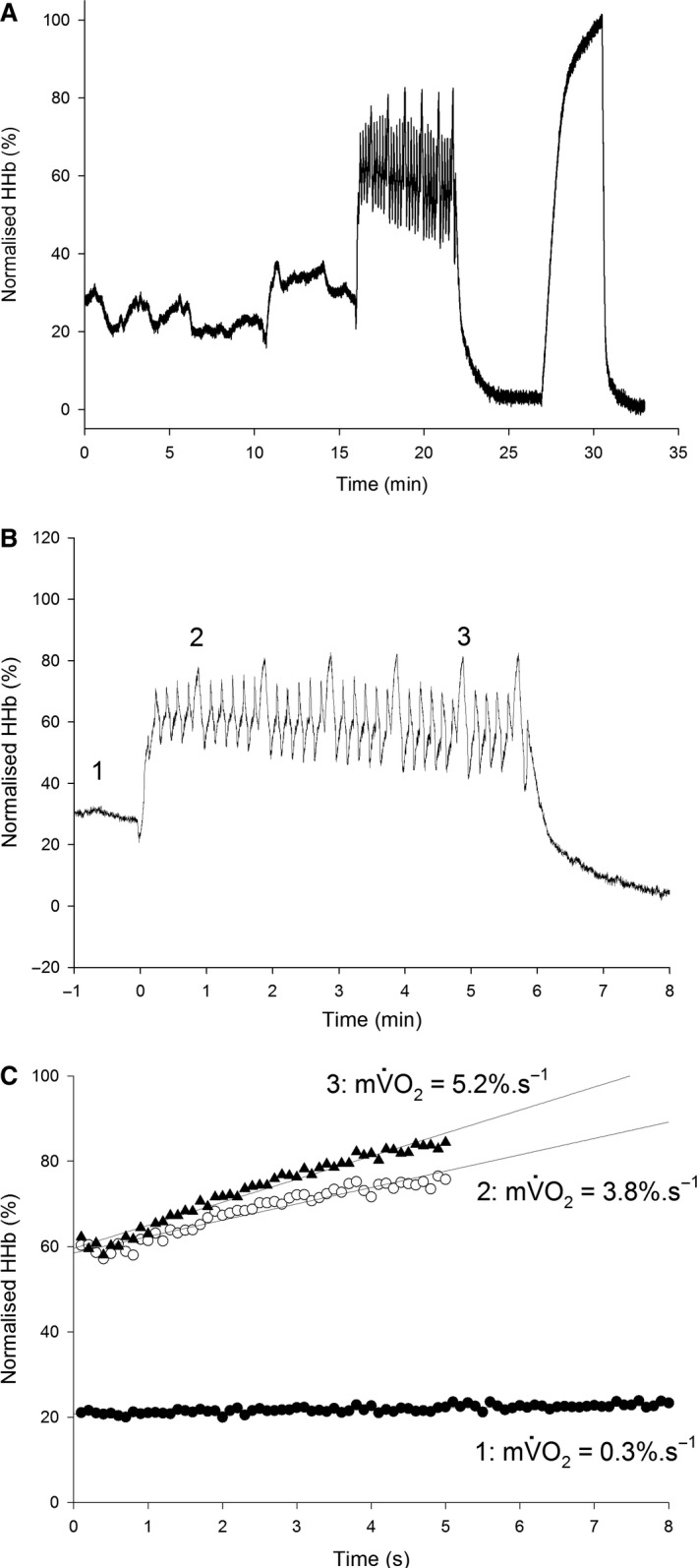
Normalized deoxygenated hemoglobin/myoglobin (HHb) response to the experimental protocol. Panel (A) shows the complete HHb record for a typical trial in a representative participant, with exercise beginning at 16 min, and the ischaemic calibration being performed at ~ 27 min. Panel (B), the HHb response to exercise in the same trial as panel (A), time aligned to the beginning of exercise. Numbers 1, 2 and 3 represent the phases used in the calculation of $m\dot{V}O_{2}$ in panel (C) (at rest, 2 min into exercise and in the last completed minute, respectively). Note the increase in $m\dot{V}O_{2}$ as exercise progresses.

$m\dot{V}O_{2}$ was determined as described in Ryan et al. (2012, 2013). $m\dot{V}O_{2}$ was calculated as the slope of the change in O_2_Hb and HHb during arterial occlusion using simple linear regression (Fig. 1). The resting $m\dot{V}O_{2}$ measurement was based on the first 8 sec (80 data points) of a 10 sec arterial occlusion, while the exercising $m\dot{V}O_{2}$ measurements were based on a 5 sec arterial occlusion (50 data points).

The NIRS data were corrected for blood volume changes as described in Ryan et al. (2012, 2013). A blood volume correction factor (β) was calculated for each data point during the arterial occlusions:

(2) $$\beta \left(t\right)=\frac{|{\text{O}}_{2}\text{Hb}\left(t\right)|}{\left(|{\text{O}}_{2}\text{Hb}\left(t\right)\left|+\right|\text{HHb}\left(t\right)|\right)}$$

where β is the blood volume correction factor, *t* is time, O_2_Hb is the oxygenated hemoglobin/myoglobin signal, and HHb is the deoxygenated hemoglobin/myoglobin signal. Each data point was corrected using its corresponding β according to equations 3 and 4, below.

(3) $$O_{2}{\text{Hb}}_{c}\left(t\right)=O_{2}\text{Hb}\left(t\right)-\left[t\text{Hb}\left(t\right)\times \left(1-\beta \right)\right]$$

(4) $${\text{HHb}}_{c}\left(t\right)=\text{HHb}\left(t\right)-\left[t\text{Hb}\left(t\right)\times \beta \right]$$

where O_2_Hb_c_ and HHb_c_ are the corrected oxygenated and deoxygenated hemoglobin/myoglobin signals, respectively; *t*Hb is the blood volume signal from the NIRS device; β is the blood volume correction factor; and *t* is time. The raw O_2_Hb signal in equation 3 was corrected by subtracting the proportion of the blood volume change attributed to O_2_Hb; while in equation 4, the raw HHb signal was corrected by subtracting the proportion of blood volume change attributed to HHb.

### Statistics

All data are presented as means ± SD. Two‐way analysis of variance (ANOVAs) with repeated measures were used to test for differences between conditions and time points, and for a condition x time interaction for MVC torque, arEMG, potentiated doublet torque, voluntary activation, variability, complexity, and $m\dot{V}O_{2}$. Data were tested for normality using the Kolmogorov–Smirnov test. The variability, complexity, and $m\dot{V}O_{2}$ measures were analyzed using means from the second minute, to account for the primary amplitude of the $\dot{V}O_{2}$ response (Burnley and Jones, 2007) and final minute before task end/failure. When main effects were observed, Bonferroni‐adjusted 95% paired‐samples confidence intervals were used to identify specific differences. The rates of change in all parameters were analyzed using Student’s paired‐samples *t*‐tests. Correlations between the rates of change in complexity and $m\dot{V}O_{2}$ were analyzed using Pearson’s product‐moment correlation (r) or, in the case of nonnormally distributed data, Spearman’s rank‐order correlation (ρ). Results were deemed statistically significant when *P* < 0.05.

## Results

### Time to task failure, MVC torque and EMG

Time to task failure at 40% MVC was 11.5 ± 5.2 min. In contrast, all participants were able to continue for 30 min without reaching task failure at 20% MVC. Both conditions resulted in significant decreases in MVC torque (*F*_1, 12_ = 120.28, *P* < 0.001; Table 1). Task failure at 40% MVC occurred when participants were no longer able to achieve the target torque (97.7 ± 22.9 N·m) despite a maximal effort. At task failure at 40% MVC, MVC torque was not significantly different from the target torque (mean difference; 95% paired samples confidence intervals (CIs): 10.2 N·m; −2.3, 18.0 N·m). At task end at 20% MVC, MVC torque remained significantly higher than the target torque (mean difference; CIs: 153.3 N·m; 128.6, 177.9 N·m) and significantly higher than at task failure at 40% MVC (mean difference; CIs: 94.3 N·m; 66.8, 121.7 N·m). The rate of decrease in MVC torque was significantly greater at 40% MVC (mean difference; CIs: 13.2 N·m; 7.5, 18.9 N·m·min^−1^; Table 1).

**Table 1:** Voluntary torque, potentiated doublet torque, voluntary activation, EMG, and $m\dot{V}O_{2}$ responses during contractions at 20% and 40% MVC.

Parameter	20% MVC	40% MVC
Target torque, N·m	49.3 ± 11.8	97.7 ± 22.9
Time to task end/failure, min	30.0 ± 0.0	11.5 ± 5.2^#^
Global fatigue		
Preexercise MVC, N·m	249.3 ± 60.7	241.5 ± 62.2
Peak MVC at task end/failure, N·m	201.7 ± 53.3^*^	107.9 ± 24.9^*,†^
Mean MVC at task end/failure, N·m	171.5 ± 51.3	91.3 ± 29.3
∆MVC/∆t, N·m·min^−1^	−1.6 ± 0.7	−14.9 ± 10.1^†^
Peripheral fatigue		
Preexercise doublet, N·m	98.2 ± 32.0	101.7 ± 28.7
Doublet at task end/failure, N·m	95.3 ± 31.6	62.8 ± 17.0^*^
% Change at task end/failure	2.3 ± 14.2	37.1 ± 11.5
∆doublet/∆t, N·m·min^−1^	−0.1 ± 0.3	−4.0 ± 3.4^†^
Central fatigue		
Preexercise VA, %	93.2 ± 3.5	94.3 ± 2.4
VA at task end/failure, %	91.4 ± 5.0	78.5 ± 10.0^*^
% Change at task end/failure	2.0 ± 3.8	16.7 ± 11.0
∆VA/∆t, %/min	−0.06 ± 0.1	−1.4 ± 1.0^†^
Surface EMG		
arEMG at task beginning, % MVC	23.0 ± 6.8	41.6 ± 8.9^†^
arEMG at task end/failure, % MVC	27.0 ± 8.7	63.0 ± 11.6^*,†^
∆arEMG/∆t, % MVC/min	0.2 ± 0.2	2.0 ± 1.3^†^
$m\dot{V}O_{2}$		
$m\dot{V}O_{2}$ at task beginning, %·sec^−1^	1.4 ± 0.8	2.7 ± 1.5^†^
$m\dot{V}O_{2}$ at task end/failure, %·sec^−1^	1.8 ± 1.1	4.0 ± 1.2^*,†^
∆$m\dot{V}O_{2}$/∆t, %·sec^−1^	0.01 ± 0.02	0.2 ± 0.08^†^

Values are means ± SD. MVC, maximal voluntary contraction; VA, voluntary activation; EMG, electromyogram; arEMG, average rectified EMG of the vastus lateralis; $m\dot{V}O_{2}$, muscle oxygen consumption; ∆, change; t, time. Task beginning values are values from 2 min into exercise, to account for primary amplitude of $\dot{V}O_{2}$ response. Symbols indicate a statistically significant difference compared to the following: *preexercise value/value at task beginning, †20% MVC.

**Figure 2:**
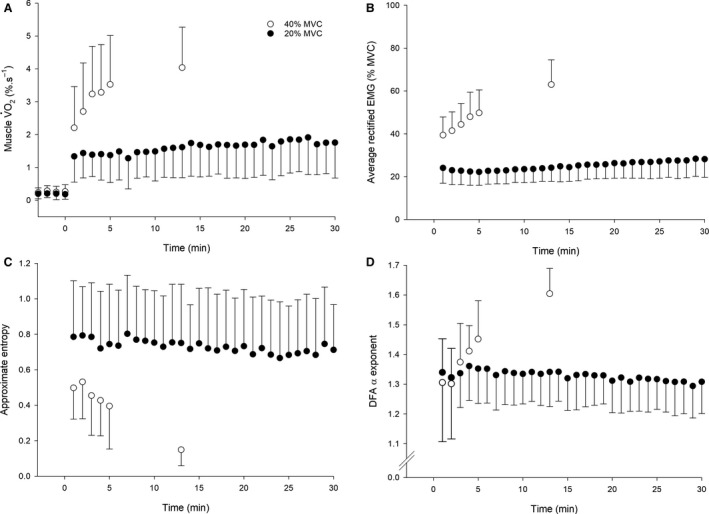
The $m\dot{V}O_{2}$ (panel A), average rectified EMG (panel B), ApEn (panel C), and DFA α exponent (panel D) responses to contractions performed at 20% MVC (black circles) and 40% MVC (white circles). Note the steady‐state response in $m\dot{V}O_{2}$ at 20% MVC, accompanied by no change in torque output complexity, in contrast to the nonsteady‐state responses in all variables at 40% MVC. In the 40% MVC trial, the last data point represents the task failure value, with the preceding value (at 5 min) being the last time point common to all participants. *N* = 13, values are means ± SD.

There was a significant condition x time effect on arEMG (*F*_1,12_ = 14.29, *P* = 0.003). The arEMG of the vastus lateralis increased over time at 40% MVC (mean difference; CIs: 21.4%; 9.0, 33.7%). The arEMG also increased at 20% MVC (mean difference; CIs: 5.0%; 0.3, 9.6%), though remained significantly lower at task end at 20% MVC compared to task failure at 40% MVC (mean difference; CIs: −35.0%; −23.0, −47.0%; Fig. 2B). The rate of increase in arEMG was significantly greater at 40% MVC (mean difference; CIs: 1.8%; 1.1, 2.5%·min^−1^; Table 1).

### Peripheral and central fatigue

There was a significant condition x time effect on potentiated doublet torque (*F*_1,11_ = 47.30, *P* < 0.001; Table 1). Potentiated doublet torque decreased significantly at 40% MVC (mean difference; CIs: −39.0 N·m; −54.4, −23.4 N·m), indicating the presence of peripheral fatigue. There was no decrease in potentiated doublet torque at 20% MVC (mean difference; CIs: −3.2; −9.6, 3.2 N·m) and the potentiated doublet torque remained significantly higher at task end at 20% MVC compared to task failure at 40% MVC (mean difference; CIs: 32.0 N·m; 16.2, 47.7 N·m). The rate of decrease in potentiated doublet torque was significantly greater at 40% MVC (mean difference; CIs: 3.9 N·m;1.8, 5.9 N·m·min^−1^; Table 1).

There was a significant condition x time effect on voluntary activation (*F*_1,11_ = 27.81, *P* < 0.001; Table 2). Voluntary activation decreased significantly at 40% MVC (mean difference; CIs: −15.7%; −24.2, −7.3%), indicating the presence of central fatigue. There was no decrease in voluntary activation at 20% MVC (mean difference; CIs: −2.1%; −5.0, 0.8%) and voluntary activation remained significantly higher at task end at 20% MVC compared to task failure at 40% MVC (mean difference; CIs: 12.8%; 4.4, 21.3%). The rate of decrease in voluntary activation was significantly greater at 40% MVC (mean difference; CIs: 1.4%.min^−1^; 0.8, 2.0%·min^−1^; Table 1).

**Table 2:** Variability, complexity and fractal scaling responses during contractions at 20% and 40% MVC.

Parameter	20% MVC	40% MVC
SD		
SD at task beginning, N·m	1.1 ± 0.2	2.4 ± 0.8^†^
SD at task failure, N·m	1.5 ± 0.4	7.3 ± 3.0^*,†^
∆SD/∆t, N·m·min^−1^	0.01 ± 0.01	0.8 ± 0.7^#^
CV		
CV at task beginning, %	2.2 ± 0.9	2.5 ± 0.8
CV at task failure, %	2.8 ± 1.3	8.2 ± 2.7^*,†^
ΔCV/Δt, %/min	0.02 ± 0.03	0.9 ± 0.7^#^
ApEn		
ApEn at task beginning	0.80 ± 0.25	0.53 ± 0.19^†^
ApEn at task failure	0.73 ± 0.25	0.15 ± 0.09^*,†^
∆ApEn/∆t	−0.003 ± 0.003	−0.05 ± 0.04^†^
DFA α		
DFA α at task beginning	1.32 ± 0.09	1.31 ± 0.18
DFA α at task failure	1.30 ± 0.10	1.60 ± 0.09^*,†^
∆DFA α/∆t	0.001 ± 0.001	0.06 ± 0.09^†^

Values are means ± SD. SD, standard deviation; CV, coefficient of variation; ApEn, approximate entropy; DFA α, detrended fluctuation analysis; ∆, change; t, time. Task beginning values are values from 2 min into exercise, to account for primary amplitude of $\dot{V}O_{2}$ response. Symbols indicate a statistically significant difference compared to the following: *value at task beginning, ^†^20%.

### Muscle oxygen consumption

There was a significant condition x time effect on $m\dot{V}O_{2}$ (*F*_1,12_ = 19.31, *P* = 0.001). After 2 min of contractions, wherein a steady state should have been observed, $m\dot{V}O_{2}$ increased significantly during the contractions at 40% MVC (mean difference; CIs: 1.3%·sec^−1^; 0.9, 1.8%·sec^−1^), but did not change during the contractions at 20% MVC (mean difference; CIs: −0.3%·sec^−1^; −0.7, 0.06%·sec^−1^; Figure 2A). $m\dot{V}O_{2}$ remained significantly lower at task end at 20% MVC compared to task failure at 40% MVC (mean difference; CIs: −2.8%·sec^−1^; −1.8, −2.7%·sec^−1^). The rate of increase in $m\dot{V}O_{2}$ was significantly greater at 40% MVC (mean difference; CIs: 0.2%·sec^−1^; 0.1, 0.2%·sec^−1^).

### Variability and complexity

The variability and complexity data are presented in Table 2. There was a significant condition x time effect on the amount of variability, as measured by the SD (*F*_1,12_ = 43.94, *P* < 0.001) and CV (*F*_1,12_ = 63.06, *P* < 0.001). Both the SD (mean difference; CIs: 4.9 N·m; 2.8, 7.0 N·m) and CV (mean difference; CIs: 5.6%; 3.7, 7.6%) increased significantly at 40% MVC; while there was no change in either at 20% MVC (SD mean difference; CIs: −0.3 N·m; −0.7, 0.01 N·m; CV mean difference; CIs: −0.6%; −1.3, 0.02%). The SD (mean difference; CIs: −5.8 N·m; −3.5, −8.2 N·m) and CV (mean difference; CIs: −5.3% −3.1, −7.6%) remained significantly lower at task end at 20% MVC compared to task failure at 40% MVC. The rates of increase in the SD (mean difference; CIs: 0.7 N·m·min^−1^; 0.3, 1.2 N·m·min^−1^) and CV (mean difference; CIs: 0.8%·min^−1^; 0.4, 1.2%·min^−1^) were significantly greater at 40% MVC.

**Figure 3:**
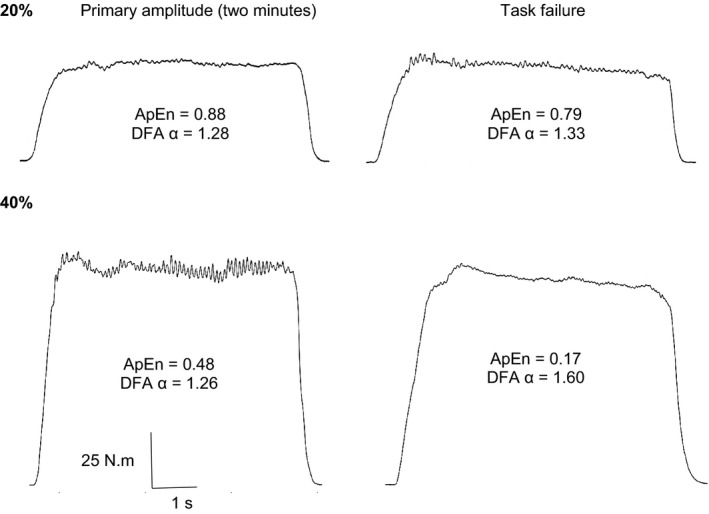
Raw torque output in a representative participant during contractions performed at 20% MVC (top two panels) and 40% MVC (bottom two panels). Note the lack of change in torque complexity in panel A, but the substantial reduction in complexity in panel B.

Example contractions from a representative participant are shown in Figure 3. There was a significant condition x time effect on the complexity of muscle torque output, as measured by ApEn (*F*_1,12_ = 63.26, *P* < 0.001) and DFA α (*F*_1,12_ = 29.47, *P* < 0.001). ApEn decreased significantly at 40% MVC (mean difference; CIs: −0.4; −0.5, −0.3), but did not change at 20% MVC (mean difference; CIs: −0.08; −0.008, 0.2). ApEn remained significantly higher at task end at 20% MVC than at task failure at 40% MVC (mean difference; CIs: 0.6; 0.4, 0.8; Fig. 2C). The rate of decrease in ApEn was significantly greater at 40% MVC (mean difference; mean CIs: 0.05; 0.03, 0.07). DFA α increased significantly at 40% MVC (mean difference; CIs: 0.28; 0.1, 0.4), but did not change at 20% MVC (mean difference; CIs: −0.01; −0.02, 0.04). DFA α remained significantly lower at task end at 20% MVC than at task failure at 40% MVC (mean difference; CIs: −0.29, −0.2, −0.4; Fig. 2D). The rate of increase in DFA α was significantly greater at 40% MVC condition (mean difference; CIs: 0.04; 0.02, 0.07).

### Correlations

**Figure 4:**
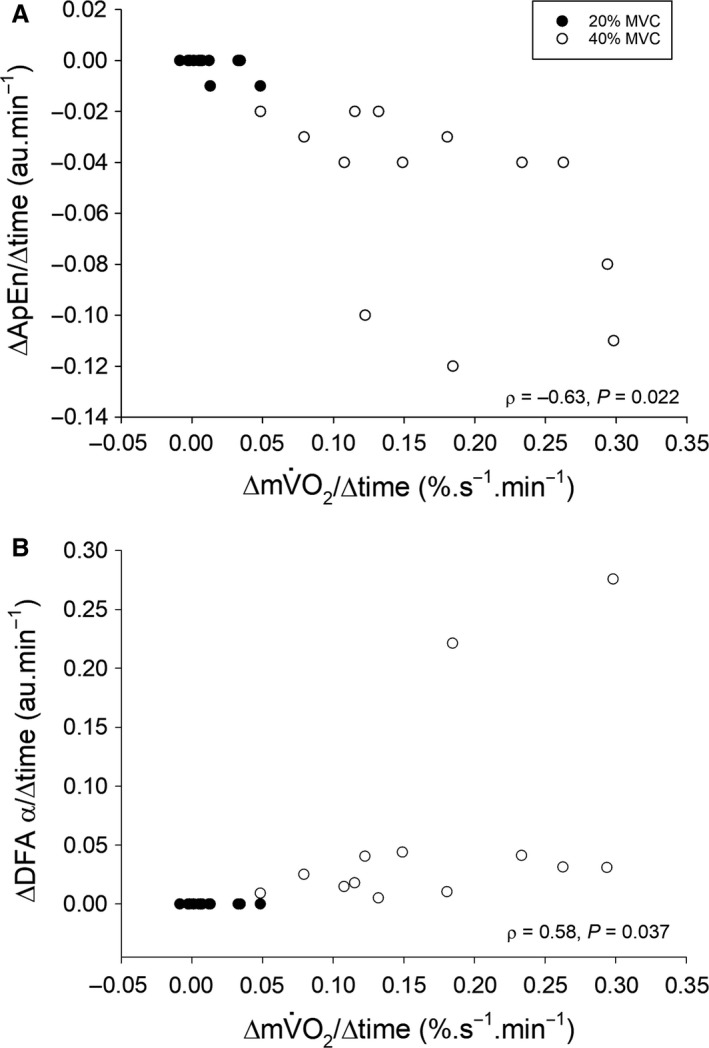
Relationship between the change in $m\dot{V}O_{2}$ and the change in ApEn and the DFA α exponent at during contractions 20% MVC (black circles) and 40% MVC (white circles). The rates of change in each variable are derived from the difference between the task failure value and the value at 2 min (the so‐called primary amplitude of the $m\dot{V}O_{2}$ response), following which a steady state should be observed. Note the limited change at 20% MVC, but larger and more variable changes at 40% MVC. Spearman’s rank correlation coefficients are given for the 40% MVC trial. See text for further details. *N* = 13 in both conditions.

The rate of change in torque complexity in the 40% MVC condition, when quantified using both ApEn and the DFA α exponent, was not normally distributed (Kolmogorov–Smirnov test, *P* < 0.05). The Spearman’s rank correlation coefficient (ρ) was therefore used in these analyses. There was a negative correlation at 40% MVC between the rates of change in ApEn and $m\dot{V}O_{2}$ (ρ = –0.63, *P* = 0.022) and a positive correlation between DFA α and $m\dot{V}O_{2}$ (*ρ* = 0.58, *P* = 0.037). Pearson’s product‐moment correlation analysis demonstrated that there were no correlations at 20% MVC between ApEn and $m\dot{V}O_{2}$ (*r* = −0.30, *P* = 0.32) and DFA α and $m\dot{V}O_{2}$ (*r* = −0.29, *P* = 0.33; Fig. 4).

## Discussion

The major novel finding of this study was the demonstration of an inverse relationship between muscle metabolic rate and muscle torque complexity. Specifically, as $m\dot{V}O_{2}$ increased during high‐intensity fatiguing contractions, there was a concomitant decrease in muscle torque complexity, with the rates of changes of these parameters being negatively correlated. In contrast, during low‐intensity contractions there were no changes in either $m\dot{V}O_{2}$ or muscle torque complexity. This is the first study to demonstrate a correlation between the fatigue‐induced fall in muscle torque complexity and the rise in muscle metabolic rate during high‐intensity contractions. This supports Seely and Macklem (2012) hypothesis that system complexity is dependent, in part, on system metabolic rate. However, as discussed in the following sections, such relationships do not imply that a change in muscle metabolic rate causes a change in torque output complexity.

### *Relationship between* $m\dot{V}O_{2}$ *and torque complexity*

This study observed that, as seen previously (Pethick et al., 2015, 2016, 2018a, 2018b), high‐intensity (40% MVC) fatiguing intermittent isometric knee extension contractions resulted in a progressive decrease in muscle torque complexity, as measured by decreased ApEn and increased DFA α (Fig. 2; Fig. 3; Table 2). In contrast, low‐intensity contractions (20% MVC) did not result in a decline in complexity, despite a small but measurable development of neuromuscular fatigue. These observations are also consistent with our previous experiments in which contractions performed below the so‐called critical torque resulted in no decrease in complexity, whereas contractions above the critical torque resulted in a decrease in complexity until task failure occurred (Pethick et al., 2016). In the present experiments, the fall in torque complexity at 40% MVC was associated with increases in both aEMG and $m\dot{V}O_{2}$, with the latter displaying the characteristic behavior of severe‐intensity exercise (i.e., above critical power or torque; Poole et al., 1988,2016; Jones and Poole, 2005). That is, beyond the first 2 min of contractions, $m\dot{V}O_{2}$ increased as a function of time until task failure. At 20% MVC, in contrast, $m\dot{V}O_{2}$ reached steady state after ~2 min (Fig. 2A). This response is characteristic of moderate‐intensity exercise (i.e., performed substantially below the critical torque; Burnley and Jones, 2007). As shown in Figure 2, the behavior of torque complexity and that of $m\dot{V}O_{2}$ at both contraction intensities was qualitatively similar, suggesting these behaviors are linked.

In interpreting the responses observed in this study, it is necessary to address the specific hypothesis of Macklem (2009) and Seely and Macklem (2012). The prediction of this hypothesis is that the degree of variability (“complexity” as we define it above) in a physiological time series is related to the energy consumption of the system producing it. In other words, time series complexity should be proportional to the ratio of the maximum metabolic rate and the prevailing metabolic rate of the system in question (Seely and Macklem, 2012). During exercise, the muscle metabolic rate is increased above rest, reducing the ratio between the operating metabolic rate and its maximum; thus, complexity will be lowest on the attainment of the maximum metabolic rate. This is consistent with the results of the present experiments, wherein torque complexity was the lowest, and $m\dot{V}O_{2}$ the highest, at task failure in the 40% MVC trial. It is important to note that in the present work, metabolic rate has been estimated using NIRS‐derived measures of $m\dot{V}O_{2}$. Thus, the maximal measurable metabolic rate using this method is the maximal $m\dot{V}O_{2}$ ($m\dot{V}O_{\text{2max}}$) not the maximal attainable rate of ATP utilization during muscle activity. Nevertheless, the present data appear to be consistent with the hypothesis that system complexity depends upon system metabolic rate (Seely and Macklem, 2012). However, the correlations between the rate of change in $m\dot{V}O_{2}$ and the rate of change in either ApEn or the DFA α exponent at 40% MVC were modest (though statistically significant, ρ = –0.63 and 0.58, respectively), implying that no more than ~35–40% of the variance in the loss of muscle torque complexity could be explained by the increase in $m\dot{V}O_{2}$.

While the above correlations tentatively support Seely and Macklem (2012) hypothesis, it should be noted that the active skeletal muscle most likely provides the highest signal‐to‐noise ratio for metabolic rate of any physiological system (at 40% MVC, $m\dot{V}O_{2}$ increase > 15‐fold above resting values in the present experiments). Thus, the correlations between system metabolic rate and system complexity reported here are strong as they are ever likely to get using the present sample size and experimental procedures, and yet they are modest. This observation suggests that either metabolic rate is but one variable among many that determine torque complexity, or that the relationships we uncovered reflect the behavior of a covariate common to both $m\dot{V}O_{2}$ and torque complexity. These possibilities are, of course, not mutually exclusive. From first principles, it is the torque requirement which determines the amplitude of the $m\dot{V}O_{2}$ response (or, more specifically, the force impulse produced by the muscle represented by torque impulse produced across the knee joint). The proximity of knee extensor torque to the so‐called critical torque then determines the temporal behavior of $m\dot{V}O_{2}$ (Jones et al., 2010; Poole et al., 2016), the development of neuromuscular fatigue (Burnley et al., 2012), and thus the loss of torque output complexity (Pethick et al., 2015) as the contractions progress. Under these circumstances, neuromuscular fatigue serves as the aforementioned covariate, since maintaining torque output in the face of fatigue requires additional motor unit recruitment. This would increase $m\dot{V}O_{2}$, but the activation of a greater proportion of the motor unit pool also diminishes the adaptability of the muscle, reflected in the loss of torque complexity.

As noted above, the rise in $m\dot{V}O_{2}$ beyond 2 min during contractions at 40% MVC accounted for less than half of the variance in the fall in knee extensor torque complexity. The rise in $m\dot{V}O_{2}$ is most likely the result of a slow component of the $m\dot{V}O_{2}$ response to high‐intensity exercise (Poole et al., 1991, 2008). The mechanism(s) responsible for the slow component remain a matter of debate, but it is thought that the recruitment of additional motor units as exercise progresses is a major contributor (Poole et al., 1991, 2008; Jones and Poole, 2005). However, it has been suggested that a $\dot{V}O_{2}$ slow component can be generated by intracellular processes reducing efficiency independently from motor unit recruitment (Zoladz et al., 2008; Vanhatalo et al., 2010). The origin of such inefficiency remains largely conjectural, but would be caused by either an increase in the ATP cost of force production or a decrease in the P:O ratio within the recruited fibres themselves. The negative influence of fatigue on cross bridge function and/or of ionic fluxes would result in an increase in the ATP, and thus O_2_, cost of force production with no change in the P:O ratio (Poole and Jones, 2012). Alternatively, muscle contraction could result in the uncoupling of oxidative phosphorylation, reducing the P:O ratio. This could occur as a consequence of proton leak in the inner mitochondrial membrane as muscle temperature rises (Willis and Jackman, 1994), or from the action of mitochondrial uncoupling proteins (Russell et al., 2002), increasing the O_2_ cost of ATP production. However, the close relationship observed between the kinetics of pulmonary and/or muscle $\dot{V}O_{2}$ and those of phosphorylcreatine (Rossiter et al., 2002) indicates that the P:O ratio is unchanged during high‐intensity exercise. Consequently, processes reducing the P:O ratio are likely to be of limited importance in the increase in $m\dot{V}O_{2}$ reported in the present study. Thus, the modest correlation between metabolic rate and complexity observed in this study could be explained, in part, by a component of the increase in $m\dot{V}O_{2}$ at 40% MVC being independent of the mechanism(s) responsible for the fatigue‐induced loss of torque complexity (see below).

We have argued previously that the loss of torque complexity associated with the fatigue process must be a consequence of a change in the ensemble behavior of the motor unit pool (either motor unit recruitment, firing rates, or both; Pethick et al., 2015). Analysis of cumulative spike trains from surface and/or intramuscular EMG recordings have consistently demonstrated a close relationship between the cumulative motor unit spike train and muscle force output in both animal and human models (Negro et al., 2009; Thompson et al., 2018). This suggests that variations in common synaptic input to the motor unit pool accounts for force fluctuations of the kind shown in Figure 3. Moreover, increases in common synaptic input, and motor unit synchronization, have been demonstrated to occur with neuromuscular fatigue (Castronovo et al., 2015), suggesting that altered muscle torque complexity is likely caused by a fatigue‐induced change in motor unit behavior. More frequent activation of a larger number of motor units must ultimately result in an increase in $m\dot{V}O_{2}$, but as detailed above, this may only account for part of the increase in $m\dot{V}O_{2}$ during high‐intensity exercise. Thus, the modest correlations between the change in $m\dot{V}O_{2}$ and that of torque complexity could be explained by the influence of fatigue on torque complexity being unique to changes in motor unit pool behavior, whereas $m\dot{V}O_{2}$ is influenced by both the increase in the energy cost of additional and more frequent motor unit activity as well as the increased O_2_ cost of processes intrinsic to the muscle fibers as the muscle fatigues.

### Limitations

In order to estimate $m\dot{V}O_{2}$ we used the methods developed by Ryan et al. (2012) in which quadriceps muscle ischemia was imposed using rapid and supra‐systolic cuff inflation applied to the proximal thigh. We also corrected the NIRS‐derived [O_2_Hb] and [HHb] signals for changes in blood volume under the probe during the measurements. Nevertheless, it is important to acknowledge the limitations inherent in this approach. First, the penetration depth of our NIRS probe is approximately 1.5 cm, meaning that we are detecting changes in a small and relatively superficial volume of the vastus lateralis. However, Koga et al. (2017) demonstrated that the kinetics of [HHb] were not different at two different depths in the rectus femoris muscle during constant load exercise, although the kinetics of [HHb] were different from those measured in the vastus lateralis. Thus, our estimation of $m\dot{V}O_{2}$ is specific to that of the vastus lateralis, which may not reflect the $m\dot{V}O_{2}$ of other quadriceps muscles. Furthermore, we had no means of validating the $m\dot{V}O_{2}$ measurements against a gold standard (such as the direct Fick method using thermodilution and femoral artery blood gases; Vøllestad et al., 1990). That said, the resting and exercising $m\dot{V}O_{2}$ measured in this study were consistent with the values measured previously (Ryan et al., 2012). Moreover, the $m\dot{V}O_{2}$ responses themselves are those which would be expected from the intensities we intended to impose: at rest, $m\dot{V}O_{2}$ was ~0.2–0.3%·sec^−1^ at both intensities; at 20% MVC $m\dot{V}O_{2}$ rose to reach a steady state at ~1.4%·sec^−1^; and at 40% MVC $m\dot{V}O_{2}$ initially increased to ~2.7%·sec^−1^ and was nonsteady state thereafter, reaching ~4.4%·s^−1^ at task failure (Fig. 2A). Taken together, these observations show that our estimates of $m\dot{V}O_{2}$ are physiologically plausible and consistent with estimates reported previously using the same techniques.

## Conclusion

In summary, this study has demonstrated experimentally, for the first time, that muscle torque complexity and muscle metabolic rate are related. Specifically, during contractions performed at 20% MVC, $m\dot{V}O_{2}$ reached a steady state after ~2 min and muscle torque complexity did not decrease, despite a small degree of peripheral and central fatigue. During contractions at 40% MVC, a significant degree of central and peripheral fatigue developed, $m\dot{V}O_{2}$ was in the nonsteady state throughout the exercise task, and muscle torque complexity progressively decreased. In addition, the rates of change in $m\dot{V}O_{2}$ and muscle torque complexity showed a significant, albeit modest, negative correlation. These data provide support for the hypothesis that system complexity depends, in part, upon system metabolic rate.
